# Supplementary material for: The Adult and Larva of a New Species of the Genus Dila (Coleoptera, Blaptinae, Blaptini) from Himalayas, with Molecular Phylogenetic Inferences of Related Genera of the Blaptini
Source: Insects. 2023 Mar 13;14(3):284. doi: 10.3390/insects14030284 (PMC10099737; doi:10.3390/insects14030284)
Supplement: Supplementary file 1 [file insects-14-00284-s001.zip › insects-2179378-supplementary.pdf]

Table S1. List of specimens used in this study with the corresponding Accession Number

No	Species	Sampling locality	Date of collection	Collector(s)	Preservation	DNA 序列(序列号/* 代表有；- 代表无)<br>COI / Cytb / 16S / 28S
1	*Nalepa yushuensis*	Shanglaxiu, Yushu, Qinghai, China	2012-VII-21	G. Ren *et al*.	Dried	ON827495/ON856244/ON818477/ON818505
2	*Nalepa yushuensis*	Jyêgu, Yushu, Qinghai, China	2019-VII-24	X. Bai *et al*.	Ethanol	ON827500/ON856231/ON818463/ON818490
3	*Nalepa yushuensis*	Chaiwei, Qamdo, Xizang, China	2018-VIII-23	X. Bai *et al*.	Ethanol	ON827506/ON856255/ON818483/ON818512
4	*Nalepa acuminata*	Jinsha, Baiyü, Sichuan, China	2016-VIII-6	X. Li *et al*.	Ethanol	---------/ON856246/ON818479/ON818508
5	*Nalepa acuminata*	Hepo, Baiyü, Sichuan, China	2016-VIII-6	X. Li *et al*.	Ethanol	---------/---------/ON856235/ON818496
6	*Nalepa polita*	Yagra, Garzê, Sichuan, China	2016-VIII-5	X. Li *et al*.	Ethanol	ON827511/---------/ON856236/ ON818498
7	*Nalepa polita*	Chowa, Dêgê, Sichuan, China	2011-VII-26	G. Ren *et al*.	Dried	---------/ON856254/ON818488/ON818517
8	*Nalepa quadrata*	G350 road, Luhuo, Sichuan, China	2021-VII-17	X. Li *et al*.	Ethanol	ON827505/---------/ON818468/ON818495
9	*Nalepa quadrata*	Simu, Luhuo, Sichuan, China	2016-VIII-3	X. Li *et al*.	Ethanol	ON827509/ON856252/ON818486/ON818515
10	*Nalepa quadrata*	Simu, Luhuo, Sichuan, China	2016-VIII-3	X. Li *et al*.	Ethanol	ON827510/ON856253/ON818487/ON818516
11	*Nalepa quadrata*	Lean, Xinlong, Sichuan, China	2016-Ⅷ-14	X. Li *et al*.	Ethanol	ON827494/ --------- / --------- /ON818504
12	*Nalepa xinlongensis*	Mari, Xinlong, Sichuan, China	2016-VIII-14	X. Li *et al*.	Ethanol	ON827498/ON856249/ON818482/ON818511
13	*Nalepa ovalifolia*	Gongya, Dêgê, Sichuan, China	2016-VIII-6	X. Li *et al*.	Ethanol	---------/ON856247/ON818480/ON818509

No	Species	Sampling locality	Date of collection	Collector(s)	Preservation	DNA 序列(序列号/* 代表有；- 代表无)
						COI / Cytb / 16S / 28S
14	*Nalepa ovalifolia*	Xindu, Luhuo, Sichuan, China	2016-VIII-6	X. Li *et al.*	Ethanol	ON827489/ON856238/ON818471/ON818500
15	*Nalepa ovalifolia*	Gyamda, Jomda, Xizang, China	2016-VIII-8	X. Li *et al.*	Ethanol	ON827488/ON856237/ON818470/ON818499
16	*Nalepa cylindracea*	Ya’ ngan, Baqen, Xizang, China	2018-VIII-21	X. Bai *et al.*	Ethanol	ON827502/ON856230/ON818465/ON818492
17	*Nalepa cylindracea*	Ya’ ngan, Baqen, Xizang, China	2018-VIII-21	X. Bai *et al.*	Ethanol	ON827503/ON856232/ON818466/ON818493
18	*Nalepa cylindracea*	Gyidoi, Riwoqê, Xizang, China	2019-VII-27	X. Bai *et al.*	Ethanol	ON827501/---------/ON818464/ON818491
19	*Nalepa cylindracea*	Saiqu, Zadoi, Qinghai, China	2012-VII-22	G. Ren *et al.*	Dried	ON827496/ON856245/ON818478/ON818506
20	*Nalepa cylindracea*	Gesang, Zadoi, Qinghai, China	2019-VII-25	X. Bai *et al.*	Ethanol	ON827499/ON856151/---------/ON818489
21	*Nalepa cylindracea*	Oyala pass, Nangqên, Qinghai, China	2019-VII-27	X. Bai *et al.*	Ethanol	ON827502/ON856230/ON818465/ON818492
22	*Nalepa cylindracea*	Gyanbê, Gonjo, Xizang, China	2016-VIII-8	X. Li *et al.*	Ethanol	ON827492/ON856241/ON818474/---------
23	*Nalepa cylindracea*	Lhatog, Chamdo, Xizang, China	2016-VIII-9	X. Li *et al.*	Ethanol	ON827493/ON856242/ON818475/ON818503
24	*Nalepa cylindracea*	Toba, Chamdo, Xizang, China	2016-VIII-9	X. Li *et al.*	Ethanol	ON827497/ON856248/ON818481/ON818510
25	*Nalepa cylindracea*	Qu’ nyido, Jomda, Xizang, China	2016-VIII-9	X. Li *et al.*	Ethanol	ON827491/ON856240/ON818473/ON818502
26	*Nalepa cylindracea*	Qu’ nyido, Jomda, Xizang, China	2016-VIII-8	X. Li *et al.*	Ethanol	ON827490/ON856239/ON818468/ON818501
27	*Nalepa cylindracea*	G214 road, Mangkang, Xizang, China	2021-VII-13	X. Li *et al.*	Ethanol	ON827504/ON856233/ON818467/ON818494
28	*Nalepa cylindracea*	Zom La shan, Mangkang, Xizang, China	2016-VIII-12	X. Li *et al.*	Ethanol	ON827508/ON856251/ON818485/ON818514

No	Species	Sampling locality	Date of collection	Collector(s)	Preservation	DNA 序列(序列号/* 代表有；- 代表无)<br>COI / Cytb / 16S / 28S
29	*Ablapsis compressipes*	Gamtog, Jomda, Xizang, China	7.VIII.2016	X. Li *et al*.	Ethanol	---------/OQ603110/---------/OQ600261
30	*Ablapsis compressipes*	Jangra, Dêgê, Sichuan, China	6.VIII.2016	X. Li *et al*.	Ethanol	OQ587587/OQ603111/---------/OQ600262
31	*Blaps brevis*	Bangoin, Xizang, China	17.VIII.2018	X. Bai *et al*.	Ethanol	OQ587577/OQ603099/OQ600379/OQ600272
32	*Blaps brevis*	Baila, Bangoin, Xizang, China	3.VIII.2019	X. Bai *et al*.	Ethanol	OQ587578/OQ603100/---------/OQ600273
33	*Blaps apicecostata*	Rongxar, Tingri, Xizang, China	27.Ⅶ.2014	G. Ren *et al*.	Ethanol	OQ587584/OQ603104/OQ600385/OQ600281
34	*Blaps apicecostata*	Xar Qu, Biru, Xizang, China	13.VIII.2015	X. Bai *et al*.	Ethanol	OQ587586/OQ603106/OQ600387/OQ600277
35	*Blaps apicecostata*	Doina, Yatung, Xizang, China	19.VIII.2015	X. Bai *et al*.	Ethanol	OQ587576/OQ603101/---------/---------
36	*Blaps holcus*	Doina, Nedong, Xizang, China	8.VIII.2014	G. Ren *et al*.	Ethanol	---------/OQ603105/OQ600386/OQ600282
37	*Blaps holcus*	Dagzê X50,Sanghu, Xizang, China	16.VIII.2018	X. Bai *et al*.	Ethanol	OQ587579/OQ603098/OQ600380/OQ600274
38	*Blaps rhynchoptera*	Ma, an, Xichang, Sichuan, China	2.VIII.2015	X. Bai *et al*.	Ethanol	OQ587585/OQ603107/OQ600388/OQ600280
39	*Blaps rhynchoptera*	Shuiwan, Mianning, Sichuan, China	1.VIII.2020	M. Ma	Ethanol	OQ587580/OQ603097/OQ600381/OQ600275
40	*Blaps rhynchoptera*	Zhao, M *et al*.(2019)	n/a	n/a	Ethanol	MK854717
41	*Blaps garzica*	Ronggai, Baiyü, Sichuan, China	5.VIII.2016	X. Li *et al*.	Ethanol	--------- /OQ603109/OQ600389/OQ600284
42	*Blaps garzica*	Mari, Xinlong, Sichuan, China	14.VIII.2016	X. Li *et al*.	Ethanol	OQ587588/OQ603112/OQ600391/OQ600263

No	Species	Sampling locality	Date of collection	Collector(s)	Preservation	DNA 序列(序列号/* 代表有；- 代表无)<br>COI / Cytb / 16S / 28S
43	*Blaps yini*	Tongmai, Nyingri , Xizang, China	17.VIII.2020	Z. Hu	Ethanol	OQ587581/---------/OQ600382/OQ600276
44	*Blaps yini*	Egong, Bome , Xizang, China	30.Ⅶ.2021	G. Ren *et al.*	Ethanol	OQ587582/OQ603102/OQ600383/OQ600278
45	*Blaps yini*	Medog, Xizang, China	11. VIII.2016	Zh.Chen	Ethanol	--------- /OQ603108/OQ600390/OQ600283
46	*Blaps yini*	Paggai, Bome , Xizang, China	1.VIII.2021	G. Ren *et al.*	Ethanol	OQ587583/OQ603103/OQ600384/OQ600279
47	*Coelocnemodes hurzensis*	Lugu Lake, Yunnan, China	3.VI.2012	H. Huang	Dried	OQ587596/OQ603119/OQ600374/---------
48	*Coelocnemodes tibialis*	Cangshan, Dali , Yunnan, China	15.VI.2011	J. Xu	Dried	---------/OQ603122/OQ600377/---------
49	*Dila bomina*	Egong, Bome , Xizang, China	31.VII.2021	G. Ren *et al.*	Ethanol	OQ587601/OQ603126/OQ600394/---------
50	*Dila bomina*	Guxiang, Bome , Xizang, China	27.VII.2009	G. Ren *et al.*	Dried	OQ587595/OQ603120/OQ600373/OQ600270
51	*Dila laevicollis*	Dangara region, Tajikistan	21.VII.2013	J. Yao	Dried	---------/OQ603121/OQ600376/---------
52	*Dila ngaria* **sp. n.**	Xiangzi Township, Tsada , Xizang, China	24.VIII.2015	G. Ren *et al.*	Ethanol	OQ587597/---------/OQ600375/OQ600271
53	*Dila ngaria* **sp. n.** larva	Xiangzi Township, Tsada , Xizang, China	24.VIII.2015	G. Ren *et al.*	Ethanol	OQ587599/OQ603124/OQ600392/---------
54	*Dila ngaria* **sp. n.**	Tuolin Township, Tsada , Xizang, China	11.VIII.2018	X. Li *et al.*	Ethanol	OQ587600/OQ603125/OQ600393/OQ600285
55	*Prosodes zarudenyi*	Poma, Zhaosu, Xinjiang, China	25.VI.2009	Z. Pan *et al.*	Dried	OQ587592/OQ603116/OQ600370/OQ600266
56	*Prosodes zarudenyi*	Poma, Zhaosu, Xinjiang, China	25.VI.2009	Z. Pan *et al.*	Dried	OQ587593/OQ603117/OQ600371/OQ600267

No	Species	Sampling locality	Date of collection	Collector(s)	Preservation	DNA 序列(序列号/* 代表有；- 代表无)
						COI / Cytb / 16S / 28S
57	*Prosodes regeli*	Nilak, Xinjiang, China	1.VI.2006	Y. Ba *et al*.	Dried	OQ587590/OQ603114/---------/OQ600268
58	*Prosodes regeli*	Tekes, Xinjiang, China	30.V.2006	Y. Ba *et al*.	Dried	OQ587594/OQ603118/OQ600372/OQ600269
59	*Prosodes* sp.	Mori Kazak, Xinjiang, China	17.VI.2016	Y. Ba *et al*.	Dried	OQ587589/OQ603113/OQ600368/OQ600264
60	*Prosodes pekinensis*	Pailu Mountain, Haiyuan, Xinjiang, China	18.VII.2009	X.Wang *et al*.	Dried	OQ587591/OQ603115/OQ600369/OQ600265
61	*Gnaptor spinimanus*	Serbia Vrsac, Vrsacko brdo	24.VII.2015	C. Chen	Dried	OQ587598/OQ603123/OQ600378/---------
62	*Gnaptor spinimanus*	Soldati, L *et al*. (2017)	n/a	n/a	Ethanol	KX398842
63	*Oodescelis oblonga*	Kurdnin, Tokkuztara, Xinjiang, China	4.VIII.2007	C. Zhang	Dried	MG993076/MG993086/MH410298/MG993063
64	*Oodescelis affinis*	Daban, Tekes, Xinjiang, China	21.V.2009	D. Sun	Dried	MG993073/MG993083/MH410299/MG993060
65	*Oodescelis emmerichi*	Huang shian, Xixia , Henan, China	18.VIII.2008	G. Ren *et al*.	Dried	MG993074/MG993084/MH185104/MG993061
66	*Oodescelis emmerichi*	Niubei liang, Zhashui , Shaanxi, China	22.VII.2011	X. Zhu *et al*.	Dried	---------/MG993074/MH185103/---------
